# Supplementary material for: Sarcopenia, Obesity, and Sarcopenic Obesity: Relationship with Skeletal Muscle Phenotypes and Single Nucleotide Polymorphisms
Source: J Clin Med. 2021 Oct 25;10(21):4933. doi: 10.3390/jcm10214933 (PMC8584842; doi:10.3390/jcm10214933)
Supplement: Supplementary file 1 [file jcm-10-04933-s001.zip › Supplementary Table S1.pdf]

Table S1. Previous associations of single nucleotide polymorphisms with muscle-related phenotypes/performance

Single nucleotide polymorphism	Phenotype	Main results	References
ACTN3 rs1815739	Muscle size<br><br>Knee extension torque<br><br>Sarcopenia<br><br>Elite sprint athlete status	XX genotype had lower mid-thigh area than RX/RR<br><br>XX genotype had lower knee torque than RR/RX<br><br>XX genotype at higher risk of sarcopenia<br><br>RR genotype overrepresented in elite sprinter group versus controls	(Zempo et al., 2010)<br><br>(Walsh et al., 2008)<br><br>(Cho et al., 2017)<br><br>(Chen et al., 2020)
ACE rs4341 (I/D)	Lean mass<br><br>Muscle strength and performance	D allele favourable for higher lean mass<br><br>II genotype associated with higher handgrip strength and jump performance among adolescents	(Charbonneau et al., 2008)<br><br>(Moran et al., 2006)
CNTF rs1800169	Knee extension and elbow flexion<br><br>Knee flexion<br><br>Knee extension<br><br>Handgrip strength	GA heterozygotes had stronger than GG homozygotes<br><br>A-allele carriers weaker than GG homozygotes in middle aged women<br><br>G allele favourable for knee strength among elderly<br><br>AA homozygotes had 3.8 kg weaker handgrip strength than G-allele carriers	(Roth et al., 2001)<br><br>(De Mars et al., 2007)<br><br>(He et al., 2020)<br><br>(Arking et al., 2006)
CNTFR rs2070802	Knee extension and flexion	T-allele carriers could produce greater torque	(De Mars et al., 2007)
ESR1 rs4870044	Sarcopenia	T-allele carriers had higher risk of sarcopenia	(Khanal et al., 2020)
FTO rs9939609	Body mass and obesity related phenotypes<br><br>Sarcopenia	A allele favoured higher BMI, obesity indices and muscle mass<br><br>AA homozygotes at higher risk of sarcopenia than T-allele carriers	(Jacobsson et al., 2012, Al-Serri et al., 2018)<br><br>(Khanal et al., 2020)
HIF1A rs11549465	Oxygen consumption capacity<br><br>Strength/power athlete status	TT associated with higher $V0_{2max}$ among elderly<br><br>Overrepresentation of TT genotype in athletes compared to controls	(Prior et al., 2003)<br><br>(Ahmetov et al., 2008, Drozdovska et al., 2013)
ID3 rs11574	Obesity-related indices	A allele associated with increment in BMI and fat mass over the time	(Svendstrup et al., 2018)
IGF1 rs35767	Body composition	CC homozygotes had greater total fat but lower lean and muscle mass	(Kostek et al., 2010)
IL6 rs1800795	Elite athlete status	Overrepresentation of G- allele among athletes	(Ruiz et al., 2010, Cenikli et al., 2016)

MTHFR rs1801131 rs1537516 rs17421511	Strength/sprint athlete status<br><br>Maximal rate of oxygen consumption	Overrepresentation of rs1801131 C allele among athletes<br><br>rs1801131 C-allele carriers had greater improvement in VO_{2max} during training	(Zarebska et al., 2014)<br><br>(Cięszczyk et al., 2016)
PTK2 rs7843014, rs7460	Exceptional longevity<br><br>Specific force	rs7843014 CC and rs7460 TT associated with longevity<br><br>AA homozygotes had greater vastus lateralis specific force in healthy population	(Garatachea et al., 2014)<br><br>(Erskine et al., 2012, Stebbings et al., 2017)
TRHR rs7832552	Lean body mass<br><br>Sarcopenia	T allele favoured greater lean body mass<br><br>C-allele carriersat higher risk of sarcopenia than TT homozygotes	(Liu et al., 2009, Lunardi et al., 2013)<br><br>(Khanal et al., 2020)
TTN rs10497520	Endurance running performance<br><br>Knee strength	T-allele carriers had better marathon performance<br><br>C-allele is associated with higher knee strength among elderly	(Stebbings et al., 2018)<br><br>(He et al., 2018)
VDR rs2228570	Muscle size<br><br>Knee strength<br><br>Muscle mass/Sarcopenia	F (C) allele associated with lower fat-free-mass<br><br>F/FF allele/genotype had lower knee strength compared to f-allele carriers<br><br>f-allele had higher risk of sarcopenia group and lower fat-free mass	(Roth et al., 2004)<br><br>(Windelinckx et al., 2007, Hopkinson et al., 2008)<br><br>(Roth et al., 2004, Walsh et al., 2016)
MSTN rs1805086	Muscle strength/muscle size	R153 allele associated with lower muscle strength<br><br>R153 allele associated with lower muscle mass among elderly women<br><br>R allele associated with higher elbow flexion torque peak power<br><br>KR heterozygotes had greater increment in bicep and quadriceps thickness than KK homozygotes post training	(Seibert et al., 2001, Corsi et al., 2002)<br><br>(González-Freire et al., 2010)<br><br>(Kostek et al., 2009, Santiago et al., 2011)<br><br>(Li et al., 2014)
COL1A1 rs1800012	Muscle strength	A-allele associated with lower handgrip and biceps strength in elderly	(Van Pottelbergh et al., 2001)
ACVR1B rs2854464<br><br>*ACVR1B* rs10783485	Muscle strength<br><br>Sprint/power athlete status<br><br>Muscle mass<br><br>Muscle strength	A allele associated with higher knee strength<br><br>A allele overrepresented in sprint and power athletes<br><br>A allele associated with higher SMM<br><br>rs10783485 C allele linked to higher knee flexion	(Windelinckx et al., 2011)<br><br>(Voisin et al., 2016)<br><br>(He et al., 2018)<br><br>(Windelinckx et al., 2011)
NOS3 rs1799983	Power athlete status<br><br>Stroke volume<br><br>Sarcopenia	T allele common among power athletes<br><br>Postmenopausal women with T-allele had higher stroke volume during dynamic exercise<br><br>T allele carriers had higher skeletal muscle mass above sarcopenic threshold	(Gómez-Gallego et al., 2009, Sessa et al., 2011, Zmijewski et al., 2018, Eider et al., 2014)<br><br>(Hand et al., 2006)<br><br>(Khanal et al., 2020)

References

1. AHMETOV, II, HAKIMULLINA, A. M., LYUBAEVA, E. V., VINOGRADOVA, O. L. & ROGOZKIN, V. A. 2008. Effect of HIF1A gene polymorphism on human muscle performance. *Bulletin of experimental biology and medicine,* 146**,** 351-353.
2. AL-SERRI, A., AL-BUSTAN, S. A., KAMKAR, M., THOMAS, D., ALSMADI, O., AL-TEMAIMI, R., MOJIMINIYI, O. A. & ABDELLA, N. A. 2018. Association of FTO rs9939609 with Obesity in the Kuwaiti Population: A Public Health Concern? *Medical Principles and Practice,* 27**,** 145-151.
3. ARKING, D. E., FALLIN, D. M., FRIED, L. P., LI, T., BEAMER, B. A., XUE, Q. L., CHAKRAVARTI, A. & WALSTON, J. 2006. Variation in the ciliary neurotrophic factor gene and muscle strength in older Caucasian women. *Journal of the American Geriatrics Society,* 54**,** 823-826.
4. CENIKLI, A., NURSAL, A., TURAL, E., POLAT, Y., TASMEKTEPLIGIL, M. & YIGIT, S. 2016. The Correlation Between Rs1800795 Variant of IL-6 and sports performance among turkish elite athletes. *International Journal of Humanities Social Sciences and Education,* 3**,** 1-5.
5. CHARBONNEAU, D. E., HANSON, E. D., LUDLOW, A. T., DELMONICO, M. J., HURLEY, B. F. & ROTH, S. M. 2008. ACE genotype and the muscle hypertrophic and strength responses to strength training. *Medicine and science in sports and exercise,* 40**,** 677.
6. CHEN, W., ZHANG, B., LI, Y. & LIANG, T. 2020. Association Between rs1815739 Polymorphism of ACTN3 Gene and Athletic Ability in Chinese Sprinters. *Journal of Science in Sport and Exercise,* 2**,** 113-119.
7. CHO, J., LEE, I. & KANG, H. 2017. ACTN3 gene and susceptibility to sarcopenia and osteoporotic status in older Korean adults. *BioMed research international,* 2017.
8. CIĘSZCZYK, P., ZARĘBSKA, A., JASTRZĘBSKI, Z., SAWCZYN, M., KOZAKIEWICZ-DROBNIK, I., LEOŃSKA-DUNIEC, A., KACZMARCZYK, M., MACIEJEWSKA-SKRENDO, A., ŻMIJEWSKI, P. & TRYBEK, G. 2016. Does the MTHFR A1298C Polymorphism Modulate the Cardiorespiratory Response to Training? *Journal of human kinetics,* 54**,** 43-53.
9. CORSI, A. M., FERRUCCI, L., GOZZINI, A., TANINI, A. & BRANDI, M. L. 2002. Myostatin polymorphisms and age-related sarcopenia in the Italian population. *Journal of the American Geriatrics Society,* 50**,** 1463-1463.
10. DE MARS, G., WINDELINCKX, A., BEUNEN, G., DELECLUSE, C., LEFEVRE, J. & THOMIS, M. A. I. 2007. Polymorphisms in the CNTF and CNTF receptor genes are associated with muscle strength in men and women. *Journal of Applied Physiology,* 102**,** 1824-1831.
11. DROZDOVSKA, S., DOSENKO, V., AHMETOV, I. & ILYIN, V. 2013. The association of gene polymorphisms with athlete status in Ukrainians. *Biology of sport,* 30**,** 163.
12. EIDER, J., FICEK, K., KACZMARCZYK, M., MACIEJEWSKA-KARŁOWSKA, A., SAWCZUK, M. & CIĘSZCZYK, P. 2014. Endothelial nitric oxide synthase g894t (rs1799983) gene polymorphism in polish athletes. *Open Life Sciences,* 9**,** 260-267.
13. ERSKINE, R. M., WILLIAMS, A. G., JONES, D. A., STEWART, C. E. & DEGENS, H. 2012. Do PTK2 gene polymorphisms contribute to the interindividual variability in muscle strength and the response to resistance training? A preliminary report. *Journal of applied physiology,* 112**,** 1329-1334.
14. GARATACHEA, N., FUKU, N., HE, Z.-H., TIAN, Y., ARAI, Y., ABE, Y., MURAKAMI, H., MIYACHI, M., YVERT, T. & VENTURINI, L. 2014. PTK2 rs7460 and rs7843014 polymorphisms and exceptional longevity: a functional replication study. *Rejuvenation research,* 17**,** 430-438.
15. GÓMEZ-GALLEGO, F., RUIZ, J. R., BUXENS, A., ARTIEDA, M., ARTETA, D., SANTIAGO, C., RODRÍGUEZ-ROMO, G., LAO, J. I. & LUCIA, A. 2009. The− 786 T/C polymorphism of the NOS3 gene is associated with elite performance in power sports. *European journal of applied physiology,* 107**,** 565-569.
16. GONZÁLEZ-FREIRE, M., RODRÍGUEZ-ROMO, G., SANTIAGO, C., BUSTAMANTE-ARA, N., YVERT, T., GÓMEZ-GALLEGO, F., REXACH, J. A. S., RUIZ, J. R. & LUCIA, A. 2010. The K153R variant in the myostatin gene and sarcopenia at the end of the human lifespan. *Age,* 32**,** 405-409.
17. HAND, B. D., MCCOLE, S. D., BROWN, M. D., PARK, J. J., FERRELL, R. E., HUBERTY, A., DOUGLASS, L. W. & HAGBERG, J. M. 2006. NOS3 gene polymorphisms and exercise hemodynamics in postmenopausal women. *International journal of sports medicine,* 27**,** 951-958.
18. HE, L., KHANAL, P., MORSE, C. I., WILLIAMS, A. & THOMIS, M. 2020. Associations of combined genetic and epigenetic scores with muscle size and muscle strength: a pilot study in older women. *Journal of Cachexia, Sarcopenia and Muscle*.
19. HE, L., VAN ROIE, E., BOGAERTS, A., MORSE, C. I., DELECLUSE, C., VERSCHUEREN, S. & THOMIS, M. 2018. Genetic predisposition score predicts the increases of knee strength and muscle mass after one-year exercise in healthy elderly. *Experimental gerontology,* 111**,** 17-26.
20. HOPKINSON, N. S., LI, K. W., KEHOE, A., HUMPHRIES, S. E., ROUGHTON, M., MOXHAM, J., MONTGOMERY, H. & POLKEY, M. I. 2008. Vitamin D receptor genotypes influence quadriceps strength in chronic obstructive pulmonary disease. *The American journal of clinical nutrition,* 87**,** 385-390.
21. JACOBSSON, J. A., SCHIÖTH, H. B. & FREDRIKSSON, R. 2012. The impact of intronic single nucleotide polymorphisms and ethnic diversity for studies on the obesity gene FTO. *Obesity Reviews,* 13**,** 1096-1109.
22. KHANAL, P., HE, L., STEBBINGS, G., ONAMBELE-PEARSON, G. L., DEGENS, H., WILLIAMS, A., THOMIS, M. & MORSE, C. I. 2020. Prevalence and association of single nucleotide polymorphisms with sarcopenia in older women depends on definition. *Scientific reports,* 10**,** 1-9.
23. KOSTEK, M. A., ANGELOPOULOS, T. J., CLARKSON, P. M., GORDON, P. M., MOYNA, N. M., VISICH, P. S., ZOELLER, R. F., PRICE, T. B., SEIP, R. L. & THOMPSON, P. D. 2009. Myostatin and follistatin polymorphisms interact with muscle phenotypes and ethnicity. *Medicine and science in sports and exercise,* 41**,** 1063.
24. KOSTEK, M. C., DEVANEY, J. M., GORDISH-DRESSMAN, H., HARRIS, T. B., THOMPSON, P. D., CLARKSON, P. M., ANGELOPOULOS, T. J., GORDON, P. M., MOYNA, N. M. & PESCATELLO, L. S. 2010. A polymorphism near IGF1 is

associated with body composition and muscle function in women from the Health, Aging, and Body Composition Study. *European journal of applied physiology,* 110**,** 315-324.

25. LI, X., WANG, S.-J., TAN, S. C., CHEW, P. L., LIU, L., WANG, L., WEN, L. & MA, L. 2014. The A55T and K153R polymorphisms of MSTN gene are associated with the strength training-induced muscle hypertrophy among Han Chinese men. *Journal of sports sciences,* 32**,** 883-891.
26. LIU, X.-G., TAN, L.-J., LEI, S.-F., LIU, Y.-J., SHEN, H., WANG, L., YAN, H., GUO, Y.-F., XIONG, D.-H. & CHEN, X.-D. 2009. Genome-wide association and replication studies identified TRHR as an important gene for lean body mass. *The American Journal of Human Genetics,* 84**,** 418-423.
27. LUNARDI, C. C., LIMA, R. M., PEREIRA, R. W., LEITE, T. K. M., SIQUEIRA, A. B. M. & OLIVEIRA, R. J. 2013. Association between polymorphisms in the TRHR gene, fat-free mass, and muscle strength in older women. *Age,* 35**,** 2477-2483.
28. MORAN, C. N., VASSILOPOULOS, C., TSIOKANOS, A., JAMURTAS, A. Z., BAILEY, M. E. S., MONTGOMERY, H. E., WILSON, R. H. & PITSILADIS, Y. P. 2006. The associations of ACE polymorphisms with physical, physiological and skill parameters in adolescents. *European journal of human genetics,* 14**,** 332.
29. PRIOR, S. J., HAGBERG, J. M., PHARES, D. A., BROWN, M. D., FAIRFULL, L., FERRELL, R. E. & ROTH, S. M. 2003. Sequence variation in hypoxia-inducible factor 1α (HIF1A): association with maximal oxygen consumption. *Physiological genomics,* 15**,** 20-26.
30. ROTH, S. M., SCHRAGER, M. A., FERRELL, R. E., RIECHMAN, S. E., METTER, E. J., LYNCH, N. A., LINDLE, R. S. & HURLEY, B. F. 2001. CNTF genotype is associated with muscular strength and quality in humans across the adult age span. *Journal of applied physiology,* 90**,** 1205-1210.
31. ROTH, S. M., ZMUDA, J. M., CAULEY, J. A., SHEA, P. R. & FERRELL, R. E. 2004. Vitamin D receptor genotype is associated with fat-free mass and sarcopenia in elderly men. *The Journals of Gerontology Series A: Biological Sciences and Medical Sciences,* 59**,** B10-B15.
32. RUIZ, J. R., BUXENS, A., ARTIEDA, M., ARTETA, D., SANTIAGO, C., RODRÍGUEZ-ROMO, G., LAO, J. I., GÓMEZ-GALLEGO, F. & LUCIA, A. 2010. The− 174 G/C polymorphism of the IL6 gene is associated with elite power performance. *Journal of science and medicine in sport,* 13**,** 549-553.
33. SANTIAGO, C., RUIZ, J. R., RODRÍGUEZ-ROMO, G., FIUZA-LUCES, C., YVERT, T., GONZALEZ-FREIRE, M., GÓMEZ-GALLEGO, F., MORÁN, M. & LUCIA, A. 2011. The K153R polymorphism in the myostatin gene and muscle power phenotypes in young, non-athletic men. *PloS one,* 6**,** e16323.
34. SEIBERT, M. J., XUE, Q. L., FRIED, L. P. & WALSTON, J. D. 2001. Polymorphic variation in the human myostatin (GDF-8) gene and association with strength measures in the women's health and aging study II cohort. *Journal of the American Geriatrics Society,* 49**,** 1093-1096.
35. SESSA, F., CHETTA, M., PETITO, A., FRANZETTI, M., BAFUNNO, V., PISANELLI, D., SARNO, M., IUSO, S. & MARGAGLIONE, M. 2011. Gene polymorphisms and sport attitude in Italian athletes. *Genetic testing and molecular biomarkers,* 15**,** 285-290.
36. STEBBINGS, G. K., WILLIAMS, A. G., HERBERT, A. J., LOCKEY, S. J., HEFFERNAN, S. M., ERSKINE, R. M., MORSE, C. I. & DAY, S. H. 2018. TTN genotype is associated with fascicle length and marathon running performance. *Scandinavian journal of medicine & science in sports,* 28**,** 400-406.
37. STEBBINGS, G. K., WILLIAMS, A. G., MORSE, C. I. & DAY, S. H. 2017. Polymorphisms in PTK2 are associated with skeletal muscle specific force: an independent replication study. *European journal of applied physiology,* 117**,** 713-720.
38. SVENDSTRUP, M., APPEL, E. V. R., SANDHOLT, C. H., AHLUWALIA, T. S., ÄNGQUIST, L. H., THUESEN, B. H., JØRGENSEN, M. E., PEDERSEN, O., GRARUP, N. & HANSEN, T. 2018. Prospective studies exploring the possible impact of an ID3 polymorphism on changes in obesity measures. *Obesity,* 26**,** 747-754.
39. VAN POTTELBERGH, I., GOEMAERE, S., NUYTINCK, L., DE PAEPE, A. & KAUFMAN, J. M. 2001. Association of the type I collagen alpha1 Sp1 polymorphism, bone density and upper limb muscle strength in community-dwelling elderly men. *Osteoporosis International,* 12**,** 895-901.
40. VOISIN, S., GUILHERME, J. P. F. L., YAN, X., PUSHKAREV, V. P., CIESZCZYK, P., MASSIDDA, M., CALÒ, C. M., DYATLOV, D. A., KOLUPAEV, V. A. & PUSHKAREVA, Y. E. 2016. ACVR1B rs2854464 is associated with sprint/power athletic status in a large cohort of Europeans but not Brazilians. *PloS one,* 11**,** e0156316.
41. WALSH, S., LIU, D., METTER, E. J., FERRUCCI, L. & ROTH, S. M. 2008. ACTN3 genotype is associated with muscle phenotypes in women across the adult age span. *Journal of Applied Physiology,* 105**,** 1486-1491.
42. WALSH, S., LUDLOW, A. T., METTER, E. J., FERRUCCI, L. & ROTH, S. M. 2016. Replication study of the vitamin D receptor (VDR) genotype association with skeletal muscle traits and sarcopenia. *Aging clinical and experimental research,* 28**,** 435-442.
43. WINDELINCKX, A., DE MARS, G., BEUNEN, G., AERSSENS, J., DELECLUSE, C., LEFEVRE, J. & THOMIS, M. A. I. 2007. Polymorphisms in the vitamin D receptor gene are associated with muscle strength in men and women. *Osteoporosis International,* 18**,** 1235-1242.
44. WINDELINCKX, A., DE MARS, G., HUYGENS, W., PEETERS, M. W., VINCENT, B., WIJMENGA, C., LAMBRECHTS, D., DELECLUSE, C., ROTH, S. M. & METTER, E. J. 2011. Comprehensive fine mapping of chr12q12-14 and follow-up replication identify activin receptor 1B (ACVR1B) as a muscle strength gene. *European Journal of Human Genetics,* 19**,** 208.
45. ZAREBSKA, A., AHMETOV, I. I., SAWCZYN, S., WEINER, A. S., KACZMARCZYK, M., FICEK, K., MACIEJEWSKA-KARLOWSKA, A., SAWCZUK, M., LEONSKA-DUNIEC, A. & KLOCEK, T. 2014. Association of the MTHFR 1298A> C (rs1801131) polymorphism with speed and strength sports in Russian and Polish athletes. *Journal of sports sciences,* 32**,** 375-382.
46. ZEMPO, H., TANABE, K., MURAKAMI, H., IEMITSU, M., MAEDA, S. & KUNO, S. 2010. ACTN3 polymorphism affects thigh muscle area. *International journal of sports medicine,* 31**,** 138-142.

47. ZMIJEWSKI, P., CIESZCZYK, P. & AHMETOV, II 2018. The NOS3 G894T (rs1799983) and-786T/C (rs2070744) polymorphisms are associated with elite swimmer status. *Biol Sport,* 35**,** 313-319.
