# Supplementary material for: Sarcopenia, Obesity, and Sarcopenic Obesity: Relationship with Skeletal Muscle Phenotypes and Single Nucleotide Polymorphisms
Source: J Clin Med. 2021 Oct 25;10(21):4933. doi: 10.3390/jcm10214933 (PMC8584842; doi:10.3390/jcm10214933)
Supplement: Supplementary file 1 [file jcm-10-04933-s001.zip › Supplementary Table S2.pdf]

Table S2. Genotype distributions of SNPs associated with sarcopenia in obese elderly women

SNPs	Sarcopenic obese (n = 77)	Non-sarcopenic obese (n = 176)
ACTN3 rs1815739	CC = 33 (42.9%)	CC = 52 (29.9%)
	CT = 29 (37.7%)	CT = 79 (45.4%)
	TT = 15 (19.5%)	TT = 43 (24.7%)
MTHFR rs1801131	GG = 10 (13.2%)	GG = 11 (6.3%)
	GT = 38 (50.0%)	GT = 76 (43.7%)
	TT = 28 (36.8%)	TT = 87 (50.0%)
MTHFR rs1537516	AA = 0 (0.0%)	AA = 3 (1.7%)
	AG = 23 (29.9%)	AG = 22 (12.6%)
	GG = 54 (70.1%)	GG = 149 (85.6%)

% indicates the distribution of genotypes within the sarcopenic obese and non-sarcopenic obese population
